# Time value of informal care of people with alzheimer’s disease in Spain: a population-based analysis

**DOI:** 10.1007/s10198-024-01713-y

**Published:** 2024-08-09

**Authors:** Vilaplana-Prieto C, Oliva-Moreno J

**Affiliations:** 1 Department of Economic Analysis, University of Murcia, Murcia, Spain; 2 Department of Economic Analysis and Finance, University of Castilla-La Mancha, Toledo, Spain; 3 CIBER de Fragilidad y Envejecimiento Saludable, Instituto de Salud Carlos III, Madrid, Spain

**Keywords:** Economic value, Social costs, Informal care, Unpaid care, Contingent valuation, Replacement method, Alzheimer’s disease

## Abstract

The aims of this paper are to estimate the monetary value of informal care for people with Alzheimer’s disease (AD) in Spain, to compare results with those obtained in 2008 and to analyse the main determinants of the time of the value of informal care. The Survey on Disabilities, Autonomy and Dependency carried out in Spain in 2020/21 was used to obtain information about disabled individuals with AD and their informal caregivers. Assessment of informal care time was carried out using two alternative approaches: the replacement method, and the contingent valuation method (willingness to pay & willingness to accept). The number of people with AD residing in Spanish households and receiving informal care rose to more than 200,000, representing an increase of 43% compared with 2008. The average number of hours of informal care per week ranged from 86 to 101 h, with an estimated value of between €31,584 - €37,019 per year per caregiver (willingness to accept) or €71,653 - €83,984 per year (replacement). The annual total number of caregiving hours ranged between 896 and 1,061 million hours, representing between 0.52 and 0.62 of GDP in 2021 (willingness to accept) or 1.19–1.40 of GDP (replacement). The level of care needs plays a central role in explaining heterogeneity in estimates. These results should be taken into account by decision-makers for long-term care planning in the coming years.

**Supplementary Information:**

The online version contains supplementary material available at 10.1007/s10198-024-01713-y.

## Introduction

Alzheimer’s disease is considered the most common cause of dementia and one of the most common neurological disorders, being one of the diseases that impose the greatest burden on societies worldwide [32]. In recent years, much progress has been made in our understanding of the disease. Interventions have been identified that could have a preventive character, but so far, the disease remains incurable and no effective treatments are available to halt its progression. [77]. Although some studies indicate that the age-adjusted prevalence may be decreasing in some countries [49, 50, 76], it is expected that the prevalence could increase sharply in the coming years, in line with population ageing [32]. This will affect countries unevenly, and those with the most aged population pyramids will suffer the greatest impact [24].

The economic studies that have been carried out on AD reflect this impact on health and also identify a high economic impact [21, 46]. Healthcare costs are generally high, but other non-health costs are much more relevant. This is especially clear in the case of patients living at home [19, 38, 70, 91], where families play a key role, in providing funding for private care services, but primarily as the main providers of care [26, 33].

The concept of informal care is not easy to define and is subject to variations depending on the moment in time and the society in which it is provided, admitting variants such as non-professional care or family care, although the three concepts mentioned are not totally interchangeable [17, 82]. Informal care is a type of non-professional service aimed at enabling these people to perform the basic and instrumental activities of daily life. Although carers may receive support from public authorities and society [16], including training, financial compensation and respite services, their activity is non-professional, and they do not have the same protection as in an employment relationship, considering fixed working hours or the right to rest and holiday entitlement [82].

Two distinctive characteristics of informal care are: (i) as it is not dependent on a budget, like healthcare or professional non-health care, it is often invisible to public decision-makers and the rest of society [18, 81, 86, 87]; (ii) although caregiving can have positive aspects, not only for the person receiving care, but also for the caregiver [1, 4, 30, 79], prolonged caregiving, if performed for long hours and without sufficient support [17], can place a significant burden on caregivers, resulting in problems in their socio-family, at work and in their health [3, 4, 30, 44, 57, 60, 67, 89].

Several studies have addressed these questions, trying to reveal the significance of the resources deployed by informal caregivers of AD patients. [53]. However, there are still few studies that attempt to estimate the economic impact of informal care, on a national scale, from the perspective of the value of the time provided by carers. We focus precisely on this issue in the case of caregivers of people with AD in Spain, and we compare the estimated figures with those of a previous study carried out using a similar methodology with data from 2008 [59]. Concerning the latter objective, it is important to make a comparison between both periods because during this period, there have been substantial changes in the long-term care (LTC) system in Spain, and consequently, although the development of the LTC system has not been without problems [61], we should expect that improvements in the provision of professional care will have resulted in less time spent on informal care [8].

Understanding the value of informal caregiving is important for the health and social care system in order to design policies that better support family caregivers and ensure their sustained involvement in the caregiving process. [47] were able to recalculate the incremental cost-effectiveness ratios of studies that included informal caregiving costs and showed that the inclusion of informal caregiving costs has a significant impact on cost-effectiveness, and that they even outweigh medical costs. After all, it is inconsistent to neglect the health effects of informal caregivers while trying to maximize health within a given health budget [86]. Likewise, in the specific case of economic evaluations of interventions in the field of Alzheimer’s disease, [63] concluded that “*Social costs can substantially modify the results of economic evaluations. Therefore*,* taking into account social costs in diseases such as Alzheimer’s can be a key element in decision-making on public funding and on the pricing of health interventions*”.

Moreover, projections of health and social spending will be unable to catch up with the needs of the population, and this may be detrimental both to the sustainability of the system and to the wellbeing of the person cared for and the caregiver [73, 92]. However, the measurement and valuation (i.e., the conversion to monetary units) of informal care remain a methodological challenge [13, 34, 75] highlight the lack of consensus and standardisation of methods, which makes it difficult to compare estimates of the costs of informal care. In the same vein, [27], conducted a review of 111 articles to identify the methods used to determine the value of informal care provided to people with dementia. Their results suggest that, although the replacement cost method was the most commonly applied, there is no consistent approach to valuing informal care in dementia.

Caring for a person requires a high involvement of the caregiver, in both the personal and the instrumental activities of daily living [52]. Our work contributes to the valuing of the caregivers’ work. Since we apply the same items as those included in the dependency assessment scale, we can express the level of dependency of the person receiving informal care by using the same categories as the Spanish LTC system (non-eligible, moderately dependent, severely dependent and highly dependent).

Our objectives in this paper are the following: (i) to estimate the value of informal care in Spain in 2021 using the contingent and replacement valuation methods; (ii) to compare the monetary value of informal care with the costs of LTC benefits received by people with AD; (iii) to compare these with previous results available for Spain for 2008 and assess whether the development of the Spanish LTC system (the SAAD) has led to a reduced burden (in terms of reduced care times) for informal carers; (iv) to examine variation in this value by characteristics of carer and recipient, using regression analysis.

Our results show that the number of hours of informal care, and consequently the monetary value of informal care, increases by 8.3% from non-eligible to moderately dependent, by 8.2% from moderately to severely dependent and by almost 17% from severely to highly dependent. This shows a two-fold increase in the rate of increase of informal care hours between severely and highly dependent. However, policymakers who have determined the monetary amounts (for cash subsidy) or hours (for home care) in the Spanish public LTC service have not taken into account these caregiving requirements. In this sense, we observe that the cash subsidy (public home care) represents only 4.2% of the value of the hours of informal care of a highly dependent person, using the replacement method (applying the public price of an hour of formal care).

Although these results alone ought to be the subject of reflection and debate, we are also assessing the implementation of the new LTC system in Spain (which started in 2007). Whereas in 2008 (when the first data were collected), the system was in its infancy, in 2021, a priori, it could be assumed to be fully developed. By contrast, our results show that the monetary value of informal care provided to people suffering from AD rose from 0.41 to 0.67% of GDP in 2008 to 1.19-1.40% in 2021. In individual terms (as a percentage of GDP per capita) it rose from 132 to 219% (2008) to 281-329% (2021). Although in the discussion section we analyse these results in more detail (as well as the possible limitations of our study), the findings should lead to a profound reflection about whether the development of the Spanish LTC system is proving sufficient to meet the growing needs associated with this disease, both for people with the disease and for caregivers. In this sense, our results and conclusions are not necessarily restricted to Spain, given that we are talking about a global problem that will affect many countries intensely in the coming years.

In Sect. 2, we present the characteristics of the new LTC system in Spain (the SAAD). In Sect. 3, we present the characteristics of the survey. In Sect. 4, we present the results for 2021 and the comparison with 2008. In Sect. 5, we discuss the results and their policy implications.

## The Spanish LTC system

The Spanish LTC system is grounded on Act 39/2006, of 14th December, on the Promotion of Personal Autonomy and Care for Dependent Persons (the SAAD, in its Spanish acronym), which universalised access to LTC services and supports (not financing), and devised an effective expansion of public funding for all Spaniards, serving as the national framework regulation. Prior to the implementation of the SAAD, subsidies were means-tested and funded by limited local government budgets [14].

Following a needs assessment, individuals are classified as ‘non-eligible’, ‘moderately dependent’, ‘severely dependent,’ or ‘highly dependent’. The ranking scale evaluates 47 tasks grouped into the following ten activities of daily living: eating and drinking, control of physical needs, bathing and basic personal hygiene, other personal care, dressing and undressing, maintaining one’s health, mobility, moving outside the home and housework. Each activity of daily living is assigned a different weight, and there is a different scale for individuals with mental illness or cognitive disability. Additionally, the evaluation considers the degree of supervision required to perform each task. The individual’s final score is the sum of the weights of the activities of daily living in which they have difficulty, multiplied by the degree of supervision required. The degree of dependency is determined by the number of points, as follows: non-eligible (less than 25 points), moderate dependency (25 to 49 points), severe dependency (50 to 74 points), and high dependency (above 74 points). Spain’s Royal Decree 504/2007, of 20th April, approved the dependency ranking scale established by Act 39/2006, of 14th December, *Promoción de la Autonomía Personal y Atención a las Personas en Situación de Dependencia*.

Those recognized as ‘dependent’ receive an ‘individual care plan,’ which identifies the type of support and care that best meets their overall care and social needs (and includes a consultation with the family). The catalogue of services includes services for preventing dependency and promoting personal autonomy, telecare, home care, daycare and night centre service and nursing homes. Each regional authority establishes quality standards, and regional authorities accredit professional services. The SAAD includes funding for day and night care centres, as well as residential care, in addition to home care assistance.

When the competent administrations cannot provide these services, the dependent person is entitled to economic benefits: (1) a service-linked financial benefit, only awarded when care is not possible through a public care provider, (2) subsidies for personal assistance to facilitate the beneficiary’s access to education and employment and (3) cash subsidies for care in the family (to reward informal caregivers). With regard to this latter benefit, it should be noted that receiving a cash subsidy is incompatible with any form of in-kind benefit, except for telecare.

In its first months of existence, the SAAD faced serious problems such as lack of definition and uncertainty in governance, planning and organizational shortcomings, political disputes, lack of recognition of the work of families, among other problems, which continued and become chronic during the first fifteen years of its existence [61, 65].The SAAD was designed during a period of economic prosperity, but a few months after its enactment, a financial crisis (the Great Recession) struck, leading to severe budget cuts and continued delays in its roll-out. More specifically, the evolution of dependency budgets in Spain has been characterised both by the spending cuts introduced in July 2012, and by the failure of Parliament to approve the general state budget bill for two consecutive years (2019 and 2020), which necessitated operating on the basis of a 2018 budget extension. Finally, in 2021 and 2022, the State approved an increase in the budget for LTC, of 23.3% more than in 2021 and twice the 2018 amount [41].

## Data and methods

The Survey on Disability, Personal Autonomy and Dependency Situations 2020 (*Encuesta de Discapacidad*,* Autonomía personal y situaciones de Dependencia 2020* - EDAD-2020) is a macro-survey carried out by the Spanish Statistics Institute whose main objective is to “*meet the demand for information from Public Administrations and numerous users*,* such as Third-Sector Social Action organisations*,* providing a statistical basis for the planning of policies aimed at people with disabilities and that enable the promotion of autonomy and the prevention of dependency situations*”. It also seeks to obtain information about the health of the carers of people with disabilities, as well as the time devoted to caring and the repercussions on their personal life, both work and leisure^1^.

The survey was conducted in two phases. In the first phase (August 2020 to January 2021), households in which people with disabilities and/or children with limitations lived were located. In the second phase (from April 2021 to October 2021), detailed information was obtained about aspects related to disability (for persons aged 6 and over), limitations (for children aged 2 to 5), services received and caregivers.

A question of great interest is whether the epidemic caused by SARS CoV-2 could have influenced the results of EDAD-2020. In this regard, while there seems to be agreement in the literature that COVID-19 was an element of risk for the mental health of caregivers [5, 15, 64, 72), the evidence is more nuanced when the focus is on the care provided. For example, [5] find that the frequency of care provision to parents increased in 2020 in most European countries. However, [72] in the case of Austria, also in 2020, note that neither the prevalence nor the intensity of informal care seemed to have altered significantly as a result of the pandemic. In relation to our results, it should be noted that in Spain, as in other European countries, the greatest impact of the pandemic in terms of mortality and social impact (confinement measures, major economic slowdown) took place in 2020 [36]. It is important to note that EDAD-2020 was conducted in two stages. In the first phase, carried out from August 2020 to January 2021, households in which people with disabilities and/or children with limitations lived were identified. Face-to-face interviews, including both caregivers and cared-for persons, were conducted between April and October 2021. At that time, although the threat of the pandemic was still present, a large part of the population had been vaccinated (prioritising the older population), the social restrictions that occurred during 2020 had decreased, and difficulties in obtaining personal care from someone outside the household were significantly reduced in Western and Southern European countries [6]. In this sense, although it cannot be affirmed that the effect of the new waves of SARS-COV-2 was neutral in terms of prevalence and intensity of informal care, that effect, if it had existed, would have been weaker than in 2020 and is not thought to have had a notable effect on the results presented in this paper.

The geographical scope of the survey is the whole Spanish national territory, using stratified two-stage sampling. The first stage units are census sections. The second stage units are the main family dwellings (110,130 dwellings). The survey does not include hospitals or facilities.

The survey collects variables such as the characteristics of the person with one or more disabilities (sex, age, nationality, marital status, studies completed, employment situation), the equipment and conditions of the dwelling, net household income, disability domains (vision, hearing, communication, mobility, etc.), type of limitations (in children aged 2 to 5 years), state of health, diagnosed illnesses, social and economic benefits received and care received. A specific questionnaire is also included for the main caregiver, asking about personal characteristics, time spent caring, main tasks performed and effect on the caregiver’s life (state of health, professional life, leisure time and family life).

The EDAD-2020 provides information about the officially recognised degree of dependency of those people who have already been assessed. However, we are interested in knowing the degree of dependency of everyone diagnosed with AD, regardless of whether they have already been officially assessed or not. The EDAD-2020 Disability Questionnaire contains a large battery of questions about the degree of difficulty in performing activities of daily living without aids and supervision, the level of support required and the impairment that has given rise to the disability (which allows the scale corresponding to mental illness to be applied). This allows a mapping of the ranking scale (Royal Decree 504/2007) to the EDAD-2020 questions. To check the reliability of this procedure, for those people who had been officially assessed, we compared the level of accredited dependency with the degree of dependency that we had assigned using the EDAD-2020. It was observed that the degree of dependency using the EDAD-2020 is higher or equal to the accredited dependency, which is plausible, since the dependency situation may have worsened after the receipt of the official accreditation.

The EDAD-2020 provides population weights corresponding to each individual, and which enable us to obtain population-level estimates. These population weights are provided through ratio estimators with a large sample size at the national level, which ensures unbiased estimates with little sampling error. Reweighting techniques (calibration) were applied according to sex, age and nationality, which allowed adjustment of the results for the deviations that occur due to the usual lack of response in some groups within the household surveys (for example, over-representation of elderly people).

The analysis of informal caregiving consists of three stages. The first is descriptive, showing the characteristics of main informal caregivers and people with AD cared. The second calculates the annual number of hours of informal care (at the individual and aggregate levels) and the value of informal care using two alternative methods, providing a comparison of the value of care in relation to average wage and average retirement benefit, Gross Domestic Product (GDP) per capita (individual level), and GDP and SAAD expenditure (aggregate level). The values of informal care provided by caregivers receiving a cash subsidy, of public home care or of care in a public daycare centre are also obtained in order to compare the estimated value with the minimum and maximum amounts of the cash subsidy, the cost of home care or the cost of place in a daycare centre. In the third stage, we examine the variation in the value of informal care via regression analysis, considering the impact of both caregiving and care recipient characteristics.

### Time assessment

The assessment of informal care time is assessed using two alternative approaches [85]. First, in the replacement or proxy good method, care time is valued by its nature as output, that is, time is valued with regard to the costs that would be incurred if the possibility of providing informal care did not exist and care was provided by professional caregivers [58]. The unit costs of the hour of care were obtained from the Ministry of Social Rights and Agenda 2030. The unit cost is €15.66 per hour (base year 2021).

The second method chosen was contingent valuation. EDAD 2020 does not included a contingent valuation scenario among the questions asked to caregivers. So, the WTA and WTP values used to assess the time spent on informal care are obtained from two previous Spanish studies carried out in the field of informal care provided to dependent people [31, 55]. The methods used in both studies are similar, which favours the joint use of both studies. In the case of the WTA, a double value of €6.4–6.9 per hour of care is used. In the case of the WTP, a double value of €3.3–5.6 per hour is used. Another method widely used in the literature, the opportunity cost method [32], could not be applied. The information contained in the EDAD-2020 does not allow us to know the use of time foregone by carers (considering time as an input). So, this approach is not applicable to our work.

Finally, we analysed the time values reported by main caregivers (maximum 24 h/day), but we also used an alternative estimate, applying a censoring consisting of considering a maximum care time of 16 h per day. This practice is common in the cost of illness studies where informal care is an important part of valued care resources [54].

### Statistical methods

We conducted a regression analysis of the weekly monetary valuation as the outcome and included the following explanatory variables: (i) care recipient characteristics (age, sex, degree of dependency), (ii) caregiver characteristics (age, sex, marital status and level of education) and (iii) size of municipality of residence. To our knowledge no previous research had focused on this particular subject. We applied non-parametric robust regression since it provides estimates that are robust to outliers and non-normality of the residuals. It works iteratively by performing OLS regression to compute case weights based on absolute residuals, and re-running the regression using these weights until convergence. We also performed sensitivity analyses using a log-transformed outcome which accounts for extreme monetary values that could be considered as outliers (results available upon request). All analyses were performed using the statistical software STATA 16.

## Results

### Population description

According to EDAD-2020, it is estimated that, in 2021, there were 239,558 people with Alzheimer’s disease in Spain, of whom almost 70% were women, with an average age of 83 years (Table 1). Most of them had primary education (more than 80%), 48% were widowed, and more than 97% were retired. As for the degree of dependency, 41% were highly dependent, 23% severely so, 13% moderately so, and 23% were non-eligible. Almost 86% of them received informal caregiving (that is, a total of 202,102 people). With regard to the type of informal care received, 58.24% received care only from co-resident caregivers, 18.50% from non-coresident caregivers and 23.26% from both co-resident and non-coresident caregivers.

Regarding informal caregivers, almost 68% were women with an average age of 60 years, 77% had primary or secondary education, 58% were married, and 67% were the daughter/son of the person being cared for. Most of them had been caring for people for more than 8 years (40%) or for between 4 and 8 years (28%), and almost 70% of them provided assistance with basic activities of daily living (BADL) as well as instrumental activities of daily living (IADL). With respect to SAAD benefits, 17.32% of informal caregivers received a cash subsidy, intended to reward their efforts in caregiving tasks, 28.81% received public home care and 7.24% received attention in a daycare centre.

**Table 1:** Description of people with AD and their informal caregivers

	People with AD (*N* = 239,558)	People with AD who received informal caregiving (*N* = 202,102)	Informal caregivers of AD patients (*N* = 202,102)
Women, %	69.76	70.88	67.76
Age (mean-SD)	82.72 (8.80)	82.97 (8.45)	59.79 (13.78)
Level of education			
Primary studies	80.16	81.67	35.62
Secondary studies	15.52	15.00	41.29
Tertiary studies	4.32	3.33	23.09
Marital status	3.94	4.03	28.57
Single			
Married	44.72	44.51	58.51
Widowed	47.87	48.72	3.72
Divorced	3.46	2.75	9.20
Relation with economic activity			
Working	0.31	0.37	35.87
Unemployed	0.47	0.37	35.67
Retired	97.17	98.17	25.34
Disabled	0.00	0.00	3.12
Missing	2.05	1.10	0.00
Degree of dependency			
Non eligible	22.83	17.40	-
Moderate	13.07	14.10	-
Severe	22.83	24.54	-
Highly	41.26	43.96	-
Receiving informal care		----	-
Only coresident informal caregiver	64.08	58.24	-
Only non-coresident informal caregiver	15.91	18.50	-
Co-resident & non-coresident	20.00	23.26	-
Relationship			
Spouse/partner	-	-	21.84
Mother/father	-	-	0.00
Daughter/son	-	-	68.70
Others	-	-	9.46
Social services (Yes)-%	49.92	52.49	-
Time caring			
Less than one year	-	-	2.84
1–2 years	-	-	10.28
2–4 years	-	-	18.38
4–8 years	-	-	28.01
More than 8 years	-	-	40.48
Help provided with:	-	-	
BADL tasks	-	-	10.43
IADL tasks	-	-	69.57
BADL & IADL	-	-	17.32
SAAD benefits	-		
Cash subsidy	-	17.32	-
Home care	-	28.82	-
Day centre	-	7.24	-
Dependency degree of AD patient with cash benefit associated to informal care received	-		-
Moderate	-	20.91	-
Severe	-	39.09	-
High		40.00	-
Dependency degree of the AD patient receiving public home care			-
Moderate		33.14	-------
Severe		35.02	--------
High		31.84	--------
Dependency degree of the AD patient receiving attention in a public day centre			
Moderate		22.28	-------
Severe		36.27	--------
High		41.45	--------

Population data

BADL: basic activities of daily living

IADL: instrumental activities of daily living

Source: Own work using EDAD-2020

Table 2 compares the basic characteristics of caregivers and care receivers for EDAD-2008 and EDAD-2020. Among care receivers, we observe a high degree of similarity in age, sex and distribution by degree of dependency, but an increase in the percentage of those who had completed secondary education (from 6.8% in 2008 to 15% in 2020). Among informal caregivers, there was a decrease in the percentage of female caregivers (from 80.2% in 2008 to 67.76% in 2020), but an increase in single caregivers (from 17.4 to 28.57%) and in the percentage of caregivers with secondary education (from 31.5 to 41.3%) or higher education (from 7.7 to 23.09%).

Between 2008 and 2020, the number of (censored) hours per week varied from 64.41 to 72.13 for non-eligible dependent, from 72.90 to 78.12 for moderately dependent, from 84.90 to 84.53 for severely dependent and from 85.21 to 98.82 for highly dependent.

**Table 2:** Comparison of caregivers’ and carereceiver’s characteristics between EDAD-2008 and EDAD-2020. Population characteristics

	People with AD		People with AD receiving informal care		Informal caregivers of AD patients	
	EDAD	EDAD	EDAD	EDAD	EDAD	EDAD
	2008	2020	2008	2020	2008	2020
Female, %	72.1	69.76	72.90	70.88	80.21	67.76
Age (mean-SD)	81.23	82.72	81.60	82.97	56.86	59.79
	(8.11)	(8.80)	(7.6)	(8.45)	(14.4)	(13.78)
Level of education						
Primary studies	91.41	80.16	91.1	81.67	60.8	35.62
Secondary studies	6.82	15.52	6.81	15.00	31.5	41.29
Tertiary studies	2.82	4.32	2.12	3.33	7.71	23.09
Marital status						
Single	5.61	3.94	5.42	4.03	17.41	28.57
Married	42.92	44.72	41.45	44.51	71.71	58.51
Widowed	50.32	47.87	52.44	48.72	5.21	3.72
Divorced	1.01	3.46	0.83	2.75	5.72	9.20
Degree of dependency						
Non eligible	21.91	22.83	16.44	17.40		-
Moderate	14.93	13.07	15.63	14.10		-
Severe	20.82	22.83	21.65	24.54		-
High	42.11	41.26	46.53	43.96		-
Weekly informal caregiving hours						
Non eligible					64.41*	72.13*
					(81.4)**	(91.27)**
Moderate					72.90*	78.12*
					(92.7)**	(96.71)**
Severe					84.90*	84.53*
					(110.7)**	(95.55)**
High					85.21*	98.92*
					(114.2)**	(110.69)**
Weekly informal caregiving hours when AD receives cash benefit associated to informal care						
Moderate						78.90* (97.68) **
Severe						86.22* (103.57) **
High						100.80* (112.90) **
Weekly informal caregiving hours of caregivers whose AD patient receives public home care						
Moderate						55.00* (64.15) **
Severe						85.53* (113.00) **
High						84.58* (109.15) **
Weekly informal caregiving hours of caregivers whose AD patient receives attention in a public day centre						
Moderate						49.00*
						(49.00) **
Severe						47.73*
						(52.4) **
High						79.60*
						(109)**

Source: Own elaboration using EDAD-2020and Peña-Longobardo and Oliva-Moreno (2015)

All figures have been computed using population sampling weights

*Weekly hours censoring the time of care to a maximum of 16 h per day

**Weekly hours without restriction

Unlike the other tables in which we show the number of daily hours of care, in Table 2 we report the number of weekly hours of care because this was the way the information was collected in EDAD-2008

**Fig. 1:**
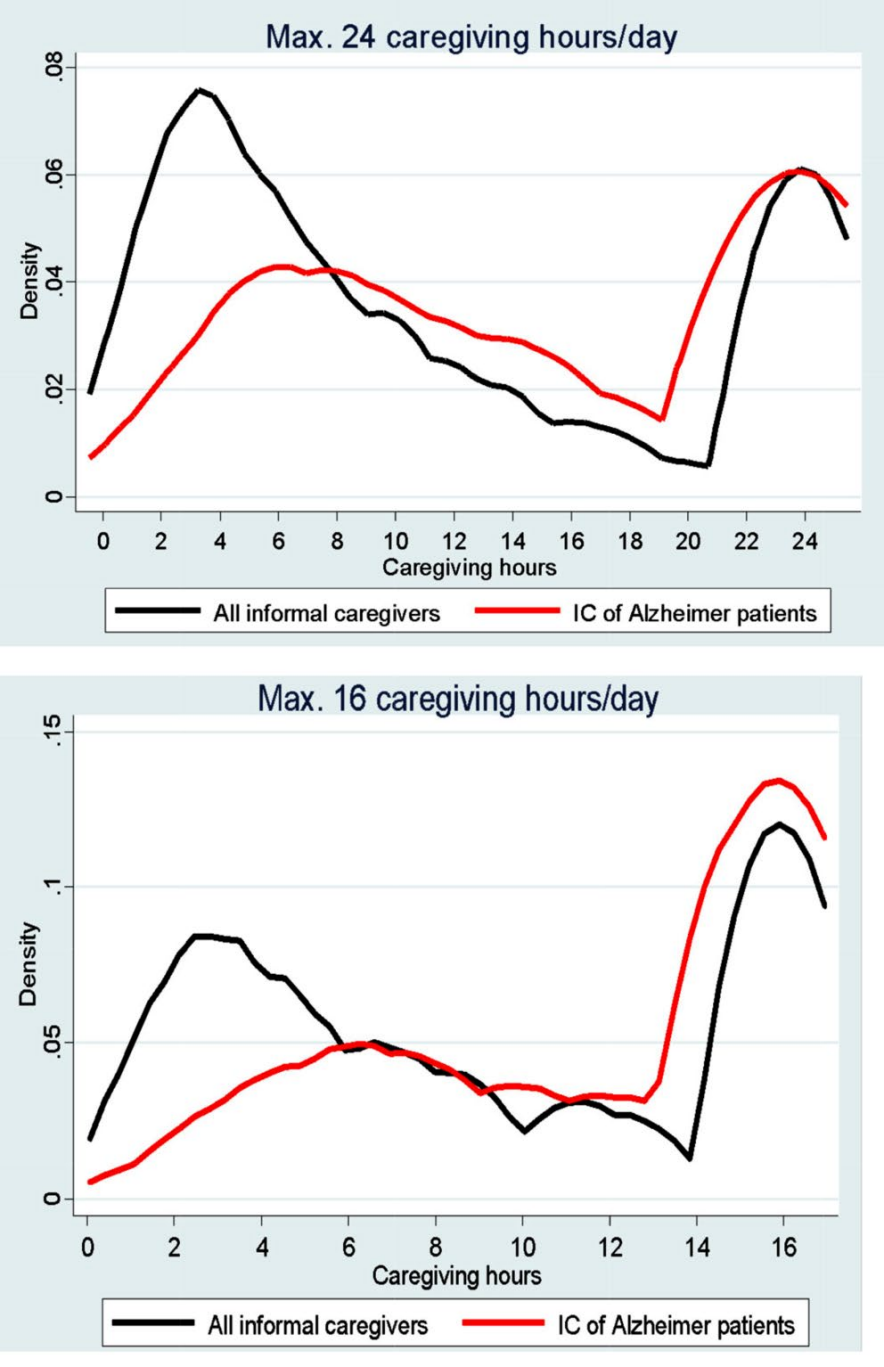
Kernel density function of informal caregiving hours. Black straight line denotes the kernel density function of all informal caregivers. Red straight line denotes the kernel density function of informal caregivers of Alzheimer patients. Upper figure considers that the maximum number of caregiving hours per day is 24, whereas lower figure restricts the maximum number of caregiving hours to 16 per day. Source: own work using EDAD-2020

Figure 1 shows the density function of the number of hours per day for the total number of informal caregivers and Alzheimer patients’ caregivers (the upper figure assumes that the maximum number of hours is 24, and the lower figure censors the maximum number of hours at 16). Both figures show a high concentration in both tails, with a large number of caregivers providing a medium or reduced number of hours per day (the greatest concentration being for total caregivers) and a very intensive group of caregivers providing the maximum number of hours possible (the greatest number, in this case, being for caregivers of patients with AD).

Table 3 shows the reported caregiving hours of AD main informal caregivers. Panel A shows the daily informal caregiving hours per caregiver. Panel B reports the annual number of informal caregiving hours per caregiver. Panel C shows the total annual number of hours of care, and panel D the percentage distribution of annual hours of care in different categories. On the left-hand side of the table the maximum number of hours is considered to be 24, while on the right-hand side it is censored at a maximum of 16 h/day.

The average number of daily hours of caregiving amounts to 15.0 (uncensored) or 11.8 h/day (censored), and most informal caregivers provide care every day. With regard to the degree of dependency, the number of uncensored (censored) daily hours of care amounts to 13.6 (11.1) for non-eligible, 14.3 (11.5) for moderately dependent, 14.0 (11.1) for severely dependent and 16.3 (14.6) for highly dependent. Annual hours of informal care per caregiver amount to nearly 5,430 h (uncensored) or 4,586 h/day (censored) and total annual hours of informal care amount to 1,097 million hours (uncensored) or 927 million hours (censored). According to the degree of dependency (and using censored hours), 20.10% of informal caregiving hours correspond to the care of non-eligible, 11.95% to moderately dependent, 20.10% to severely dependent and 47.85% to highly dependent individuals.

**Table 3:** Informal caregiving hours

	Max. 16 h/day	Max. 24 h/day
**Panel A: Daily informal caregiving hours for average caregiver**		
Days per week	6.77	6.77
	(0.81)	(0.81)
Hours per day	11.82	15.01
	(4.83)	(8.05)
Hours per week	81.14	103.17
	(34.50)	(56.45)
Dependency degree: hours per day		
Non eligible	11.06	13.64
	(5.05)	(8.09)
Moderate	11.49	14.26
	(4.47)	(7.71)
Severe	11.06	13.99
	(5.27)	(8.43)
High	14.57	16.25
	(4.55)	(7.83)
**Panel B: Annual informal caregiving hours for caregiver**		
Non eligible	3,904	4,815
Moderate	4,056	5,034
Severe	3,904	4,939
High	5,143	5,736
Average	4,470	5,281
**Panel C: Annual informal caregiving hours for total caregivers (million)**		
Non eligible	180.14	222.16
Moderate	107.14	132.97
Severe	180.14	227.87
High	428.89	478.34
Total	896.31	1,061.34
**Panel D: Distribution of annual informal caregiving hours (%)**		
Non eligible	16.97	20.93
Moderate	10.09	12.53
Severe	16.97	21.47
High	40.41	45.07

Panel A shows daily informal caregiving hours

Panel B shows the annual number of informal caregiving

Panel C shows the annual number of informal caregiving hours at population level

Panel D show the percentual distribution of yearly informal caregiving hours

Left part of the table considers that the maximum number of caregiving hours is 24. Right part of the table considers that the maximum number of caregiving hours is 16, thus all caregivers who report a number of daily hours of care greater than 16 are censored at 16 h

All figures have been computed using population sampling weights

Source: own work using EDAD-2020

### Economic assessment of informal caregiving time

#### Valuation of informal caregiving hours using contingent valuation method

Table 4 shows the annual valuation of informal caregiving according to the contingent valuation method. For each degree of dependency, the WTP (€3.3/hour) and WTA (€6.9 /hour) are shown according to the hourly price obtained by [31] and [55] respectively, per caregiver and for the total number of caregivers, whether the maximum number of hours of care is limited to 16 h/day or whether a maximum of 24 h/day is allowed.

Complementary results of the contingent valuation using WTP (€5.6/hour) [31] and WTA (€6.4/hour) [55] are shown in Table A1 in the Appendix. For better comparability of the monetary amounts, we have obtained the percentage that the individual valuation represents with respect to average wage, average retirement benefit, GDP per capita, and the valuation of the total number of caregivers with respect to GDP^2^ and with respect to SAAD expenditure on dependency^3^.

At the individual level and using WTP’s [55] shadow price, informal care valuation ranges between €12,480/year (non-eligible: NE), €13,517/year (moderately dependent: MD), €14,626/year (severely dependent: SD) and €17,099/year (highly dependent: HD), and according to [31]’s WTA between €21,094/year (NE), €22,846/year (MD), €24,720/year (SD) and €28,899/year (HD). Using the WTA, the valuation ranges between €23,921/year (NE) and €32,772€/year (HD) when we use [55]’s price and between €25,962/year (NE) and €35,568/year (HD) when we use [31]’s shadow price. Consequently, the monetary value of informal care, increases by 8.3% from non-eligible to moderately dependent, by 8.2% from moderately to severely dependent and by almost 17% from severely to highly dependent.

One of the ways to measure the relevance of the value of care is to compare it with the purchasing power of Spaniards. As indicators of this purchasing power, we chose the average wage (€25,896.82/year) and the average retirement pension (€14,277 €/year) in 2021. The value of informal care (using censored hours and average hours) represents between 59% (WTP) and 122% (WTA) of the average wage, and between 106% (WTP) and 221% (WTA) of the average retirement pension. [Complementarily, Table A1 in the Appendix shows the valuations using [31]’s WTP and [55]’s WTA].

As a percentage of GDP per capita, the valuation of informal care for an HD individual ranges between 67.1% and 113.3% (WTP) and between 128.5% and 139.5% (WTA). Overall, the valuation of informal care (the sum of NE, MD, SD and HD) ranges between €3,033 and €5,127 million (WTP) and between €5,184 and €6,310 million (WTA). As a percentage of Spain’s GDP, informal care represents between 0.25% and 0.42% (WTP) and between 0.48% and 0.52% (WTA). Finally, compared to the annual expenditure of the SAAD in 2021, informal care hours represent between 31.3% and 52.8% (WTP) and between 59.9% and 65% (WTA).

**Table 4:** Valuation of informal care using contingent valuation method

	Valuation of annual caregiving hours for all informal caregivers			
	Censored hours (max. 16 h/day)		Not censored hours (max. 24 h/day)	
	WTP	WTA	WTP	WTA
Euros				
NE	12,480	25,962	15,792	32,851
MD	13,517	28,118	16,734	34,809
SD	14,626	30,425	17,398	36,191
HD	17,099	35,568	19,152	39,840
Average	15,183	31,584	17,796	37,019
**Average valuation with respect to average wage (2021)**				
Average	59%	122%	69%	143%
**Average valuation with respect to average retirement benefit (2021)**				
Average	106%	221%	125%	259%
**Percentage with respect to per capita GDP (2021)**				
NE	48.9%	101.8%	61.9%	128.8%
MD	53.0%	110.3%	65.6%	136.5%
SD	57.4%	119.3%	68.2%	141.9%
HD	67.1%	139.5%	75.1%	156.2%
Average	59.5%	123.9%	69.8%	145.2%
	**Valuation of annual caregiving hours for all informal caregivers**			
	**Censored hours (max. 16 h/day)**		**Not censored hours (max. 24 h/day)**	
	**WTP**	**WTA**	**WTP**	**WTA**
NE	575.8	1,197.9	728.7	1,515.7
MD	357.0	742.7	442.0	919.5
SD	674.8	1,403.8	802.7	1,669.8
HD	1,425.8	2,965.9	1,597.1	3,322.2
All	3,033.5	6,310.3	3,570.5	7,427.2
**Percentage with respect to GDP (2021)**				
NE	0.05%	0.10%	0.06%	0.13%
MD	0.03%	0.06%	0.04%	0.08%
SD	0.06%	0.12%	0.07%	0.14%
HD	0.12%	0.25%	0.13%	0.28%
All	0.25%	0.52%	0.30%	0.62%
**Percentage with respect to total expenditure of the SAAD (2021)**				
NE	5.9%	12.3%	7.5%	15.6%
MD	3.7%	7.7%	4.6%	9.5%
SD	7.0%	14.5%	8.3%	17.2%
HD	14.7%	30.6%	16.5%	34.2%
All	31.3%	65.0%	36.8%	76.5%

NE: non eligible, MD: moderate dependent; SD: severe dependent; HD: highly dependent

WTP: willingness to pay; WTA: willingness to accept

WTP (Garrido-García et al., 2015) and WTA (Oliva-Moreno et al., 2019)

GDP (2021): 1,222,290 million €. Source: INE (*INEbase / Economía /Cuentas económicas /Contabilidad nacional trimestral de España: principales agregados (CNTR) / Resultados*)

GDP per capita (2021): 25,498 €

Expenditure of the SAAD (2021): 9,704,647,135.52 €. Source: *7e8ae15d-0915-ab4e-6c46-ccb0f1f3e8e2 (imserso.es)*

Average wage (2021): 25,896 €/year

Average retirement benefit (2021): 14,277 €/year

#### Valuation of informal caregiving hours using replacement method

Table 5 shows the results of the assessment of informal care according to replacement cost, both at the individual level and for all caregivers, considering a maximum of 16 h/day or 24 h/day. As in the previous table, we also report the individual valuation as a percentage of average wage, of average retirement benefit, and of GDP per capita, and the valuation of all caregivers as a percentage of GDP and of total SAAD expenditure on dependence.

Using the replacement method, at the individual level the value of informal care amounts to €58,898/year (NE), €63,789/year (MD), €69,024/year (SD) and €80,692/year (HD), which represent 231%, 250.2%, 270.7% and 316.4% respectively of per capita GDP. The value of informal care (using censored hours) was 2.77 times higher than the average salary and 5 times higher than the average retirement pension in 2021. For informal caregivers as a whole, it amounted to €14,316 million/year, which represented 1.19% of the GDP and 147.5% of the expenditure on dependency in 2021.

**Table 5:** Valuation of informal care using replacement method

	Valuation of annual in for a single informal caregiver (Euros)	
	Censored hours (max. 16 h/day)	Not censored hours (max. 24 h/day)
NE	58,898	74,527
MD	63,789	78,969
SD	69,024	82,105
HD	80,692	90,385
Average	71,653	83,984
**Average valuation with respect to average wage (2021)**		
Average	277%	324%
	**Average valuation with respect to average retirement benefit (2021)**	
Average	502%	588%
**Percentage with respect to per capita GDP (2021)**		
NE	231.0%	292.3%
MD	250.2%	309.7%
SD	270.7%	322.0%
HD	316.4%	354.5%
Average	281.0%	329.3%
	**Valuation of annual caregiving hours for all informal caregivers (Million €)**	
	**Censored hours (max. 16 h/day)**	**Not censored hours (max. 24 h/day)**
NE	2,717.6	3,438.7
MD	1,685.0	2,086.0
SD	3,184.8	3,788.3
HD	6,728.7	7,536.9
All	14,316.0	16,849.9
**Percentage with respect to GDP (2021)**		
NE	0.23%	0.28%
MD	0.14%	0.17%
SD	0.26%	0.31%
HD	0.56%	0.62%
All	1.19%	1.40%
**Percentage with respect to expenditure SAAD (2021)**		
NE	28.0%	35.4%
MD	17.4%	21.5%
SD	32.8%	39.0%
HD	69.3%	77.7%
All	147.5%	173.6%

NE: non eligible, MD: moderate dependent; SD: severe dependent; HD: highly dependent

WTP: willingness to pay; WTA: willingness to accept

GDP (2021): 1,222,290 million €. Source: INE (*INEbase / Economía /Cuentas económicas /Contabilidad nacional trimestral de España: principales agregados (CNTR) / Resultados*)

GDP per capita (2021): 25,5498€

Expenditure of the SAAD (2021): 9,704,647,135.52 €. Source: *7e8ae15d-0915-ab4e-6c46-ccb0f1f3e8e2 (imserso.es)*

Average wage (2021): 25,896 €/year

Average retirement benefit (2021): 14,277 €/year

#### Comparison of valuation of informal care with cost of SAAD benefits

As a complementary exercise, we analysed the different benefits granted by the SAAD to informal caregivers (which are not compatible with each other). The EDAD-2020 questionnaire reveals whether a dependent person receives a cash subsidy to help to pay for the care received from his/her informal caregiver, for public home care or for a place in a public daycare centre. For this subsample of informal caregivers, we have valued the hours of care using the above-mentioned methods.

##### Cash subsidy

Table 6 shows, for each degree of dependency, the valuation of the care provided by caregivers who receive a cash subsidy using the contingent valuation method (WTP according to [55]; WTA according to [31]) and the replacement method. In addition to the monetary valuation, the percentage of the cash subsidy corresponding to the degree of dependency of the person being cared for (minimum and maximum) is shown. [Complementarily, Table A2 in the Appendix shows the valuations using [31]’s WTP and [55]’s WTA].

As shown in Table 2, the number of weekly hours of informal care (max. 16 h/day) amounts to 78.90 for non-eligible, 86.22 for SD and 100.80 for HD, and the annual number of informal caregiving hours amounts to 5,256 (HD), 4,496 (SD) and 4,114 (MD) (Table 6). According to the contingent valuation method, the value of informal care for an HD individual ranges between €17,441 and €29,477/year (WTP) and between €33,428 and €36,279/year (WTA) for HD. According to the replacement method, the value of informal care hours amounts to €82,306/year (HD), €70,404/year (SD) and €64,427/year (MD).

Using the most generous contingent valuation ([31]’s WTA), the maximum cash subsidy represents only 9.6% (HD), 7.8% (SD) and 6.5% (MD) of the value of care. Using the most conservative contingent valuation ([55]s WTP), the cash subsidy represents 20% (HD), 16.2% (SD) and 13.4% (MD) of the value of informal care. Using the replacement method, the maximum cash subsidy represents 4.2% (HD), 3.4% (SD) and 2.8% (MD) of the value of informal care. Taking into account that there is a co-payment commensurate with the beneficiary’s financial means, the average cash subsidy finally received is usually about 86%^4^ of the maximum cash subsidy (for HD and SD). Thus, the amount of the co-payment is only equivalent to a small part of the value of informal care.

**Table 6:** Valuation of annual caregiving hours of informal caregiver receiving cash subsidy

	Contingent valuation method				Replacement method	
	Censored hours (max. 16 h/day)		Not censored hours (max. 24 h/day)		Censored hours (max. 16 h/day)	Not censored hours (max. 24 h/day)
	WTP	WTA	WTP	WTA		
MD	13,652	28,399	16,901	35,157	64,427	79,759
SD	14,919	31,033	17,920	37,276	70,404	84,568
HD	17,441	36,279	19,535	40,637	82,306	92,192
**Percentage of minimum cash subsidy with respect to valuation of informal care (2021)**						
	**Contingent valuation method**				**Replacement method**	
	**Censored hours** **(max. 16 h/day)**		**Not censored hours** **(max. 24 h/day)**		**Censored hours** **(max. 16 h/day)**	**Not censored hours (max. 24 h/day)**
	**WTP**	**WTA**	**WTP**	**WTA**		
MD	13.4%	6.5%	10.9%	5.2%	2.8%	2.3%
SD	21.6%	10.4%	18.0%	8.7%	4.6%	3.8%
HD	26.7%	12.8%	23.8%	11.4%	5.7%	5.0%
**Percentage of maximum cash subsidy with respect to valuation of informal care (2021)**						
	**Contingent valuation method**				**Replacement method**	
	**Censored hours** **(max. 16 h/day)**		**Not censored hours** **(max. 24 h/day)**		**Censored hours** **(max. 16 h/day)**	**Not censored hours (max. 24 h/day)**
	**WTP**	**WTA**	**WTP**	**WTA**		
MD	13.4%	6.5%	10.9%	5.2%	2.8%	2.3%
SD	16.2%	7.8%	13.5%	6.5%	3.4%	2.9%
HD	20.0%	9.6%	17.9%	8.6%	4.2%	3.8%

MD: moderate dependent; SD: severe dependent; HD: highly dependent

WTP: willingness to pay; WTA: willingness to accept

WTP (Garrido-García et al., 2015) and WTA (Oliva-Moreno et al., 2019)

Minimum cash subsidy: 1,836 €/year (MD); 2,419.08 €/year (SD); 3,488.76 €/year (HD)

Maximum cash subsidy: 1,836 €/year (MD); 3,225.48 €/year (SD); 4,651.68 €/year (HD)

##### Home care

The next step is the computation of the value of informal care for caregivers whose patients receive in-kind benefits (home care and day care). Consistently, the number of hours of care for a caregiver who attends an HD patient and receives a cash benefit is 19% higher than that of a caregiver of an HD patient receiving home care and 26% higher than that of a caregiver of an HD patient receiving public centre care (using censored hours) (see Table 2).

The hours of informal care received by AD patients receiving public home care are 8.12 h/day (MD), 12.45 h/day (SD) and 12.08 h/day (HD) if we set a maximum of 16 h/day, and amount to 9.48 h/day (MD), 16.45 h/day (SD) and 15.59 h/day, with a maximum of 24 h/day. Table 7 shows, for each degree of dependency, the value of this informal care using the contingent valuation method (WTP according to [55]; WTA according to [31]) and the replacement method. [Complementarily, Table A3 in the Appendix shows the valuations using [31]’s WTP and [55]’s WTA].

The value of informal care (using censored hours) amounts to €9,517/year (MD), €14,799 /year (SD) and €14,645/year (HD) using WTP and to €19,796/year (MD), €30,784/year (SD) and €30,444/year (HD) using WTA. The number of hours of home care received is conditioned by the degree of dependency: between 8 and 20 h/month for MD; between 21 and 45 h/month for SD and between 46 and 70 h/month for HD^5^. However, the EDAD-2020 does not provide information about the number of hours received, so we have chosen the midpoint of each interval (14, 33 and 58 h/month respectively for MD, SD and HD). The cost of formal care received represents between 3.81% (HD) and 24.27% (MD) of the value of informal care using the replacement method, and between 7.27% (HD) and 51.23% (MD) using WTA.

**Table 7:** Valuation of annual caregiving hours of informal caregiver when AD patient receives public home care

	Contingent valuation method				Replacement method	
	Censored hours (max. 16 h/day)		Not censored hours (max. 24 h/day)		Censored hours (max. 16 h/day)	Not censored hours (max. 24 h/day)
	WTP	WTA	WTP	WTA		
MD	9,517	19,796	11,100	23,091	44,911	52,385
SD	14,799	30,784	19,552	40,672	69,840	92,271
HD	14,635	30,444	18,885	39,285	69,067	89,124
**Percentage cost of public home care with respect to valuation of informal care (2021)**						
	**Contingent valuation method**				**Replacement method**	
	**Censored hours** **(max. 16 h/day)**		**Not censored hours** **(max. 24 h/day)**		**Censored hours** **(max. 16 h/day)**	**Not censored hours (max. 24 h/day)**
	**WTP**	**WTA**	**WTP**	**WTA**		
MD	114.53%	55.06%	98.19%	47.20%	24.27%	20.81%
SD	41.90%	20.14%	31.72%	15.25%	8.88%	6.72%
HD	17.98%	8.64%	13.93%	6.70%	3.81%	2.95%

MD: moderate dependent; SD: severe dependent; HD: highly dependent

WTP: willingness to pay; WTA: willingness to accept

WTP (Garrido-García et al., 2015) and WTA (Oliva-Moreno et al., 2019)

Public price of home care hour: 15.66 €/hour

MD receive between 8 and 20 h/month; SD between 21 and 45 €/month; HD between 46 and 70 €/month. In order to calculate the public cost of home care according to the degree of dependency, we have assumed that the patient receives the average number of hours corresponding to his or her degree of dependency

##### Day centre

In the case of people with AD attending a daycare centre, we observe that the number of hours of informal care is lower, both for censored hours (7.24 h/week for MD; 7 for SD; 11.67 for HD) and for uncensored hours (7.44 for MD; 7.98 for SD; 15.98 for HD). Table 8 (and A4) show, for each degree of dependency, the value of informal care using the contingent valuation method (WTP according to [55]; WTA according [31]) and the replacement method. [Complementarily, Table A4 in the Appendix shows the valuations using [31]’s WTP and [55]’s WTA].

Considering censored hours, the value of informal care amounts to €8,478/year (MD), €8,259/year (SD) and €13,773/year (HD) using WTP, and €17,636/year (MD), €17,181/year (SD) and €28,650/year (SD) using WTA. According to the replacement method, the value of care amounts to €40,011/year (MD), €38,977/year (SD) and €64,998/year (HD). Then we compare these valuations with the cost of a public place in a daycare centre in 2021 (€9,309.21/year)^6^. The cost of the public place represents between 14.32% (MD) and 23.37% (HD) of the value of informal care using the replacement method and between 32.49% (MD) and 52.78% (MD) using WTA.

**Table 8:** Valuation of annual caregiving hours of informal caregiver when AD patient receives attention in a public day centre

	Contingent valuation method				Replacement method	
	Censored hours (max. 16 h/day)		Not censored hours (max. 24 h/day)		Censored hours (max. 16 h/day)	Not censored hours (max. 24 h/day)
	WTP	WTA	WTP	WTA		
MD	8,478	17,636	8,478	17,636	40,011	40,011
SD	8,259	17,181	8,259	18,860	38,977	42,788
HD	13,773	28,650	13,773	39,232	64,998	89,005
**Percentage cost of public place in day centre with respect to valuation of informal care (2021)**						
	**Contingent valuation method**				**Replacement method**	
	**Censored hours** **(max. 16 h/day)**		**Not censored hours** **(max. 24 h/day)**		**Censored hours** **(max. 16 h/day)**	**Not censored hours (max. 24 h/day)**
	**WTP**	**WTA**	**WTP**	**WTA**		
MD	109.80%	52.78%	109.80%	52.78%	23.27%	23.27%
SD	112.71%	54.18%	112.71%	49.36%	23.88%	21.76%
HD	67.59%	32.49%	67.59%	23.73%	14.32%	10.46%

MD: moderate dependent; SD: severe dependent; HD: highly dependent

WTP: willingness to pay; WTA: willingness to accept

WTP (Garrido-García et al., 2015) and WTA (Oliva-Moreno et al., 2019)

Public price of place in day centre (2021): 9,309.21 €/year

### Comparison of informal care valuations 2008–2021

Comparing our estimation with that of [60] for 2008, we observe that the number of home-based people with AD increased by 43% between 2008 and 2021, and the number of people who received informal care also increased, by a similar amount (from 141,617 to 202,102). Censoring the maximum number of care hours per day at 16, the number of annual hours of individual-level caregiving increased by 7.7% (from 4,151 h/year to 4,470 h/year), but only by a mere 0.34% (from 5,263 h/year to 5,281 h/year) with uncensored data. Thus, the estimated total informal care time in 2008 ranged between 595 and 745 million hours, with and without censorship respectively.

In 2021, care time ranged between 927 and 1,097 million hours, with and without censorship respectively, representing an increase by 56% using censored hours (from 595 to 927 million hours) and by 47% using uncensored hours (from 745 to 1,097 million hours). This is related to the larger increase in the percentage of carers providing up to 16 h of care per day (from 42.87% in 2008 to 59.04% in 2020) and between 21 and 24 h per day (from 89.64 in 2008 to 91.30% in 2020) (see Table 9). Regarding the valuation of informal care hours (and focusing on the estimates using censored hours) we observe that: (i) using the replacement method it increased from 0.41 to 1.19% of GDP or from 132 to 281% of GDP per capita; (ii) according to the contingent valuation method (WTA), it increased from 0.24 to 0.52% of GDP and from 77 to 124% of GDP per capita. So the cost of informal care (with respect to GDP) had been multiplied by 2.9 (2.2) according to the replacement (contingent valuation) method.

Given that total SAAD spending represented 0.80% of GDP in 2021, the monetary value of informal care was between 149% and 175% of the SAAD budget. As a further reference, average LTC spending in EU-21 countries in 2019 was 1.74% of GDP and in the Nordic countries it reached figures above 2% of GDP (European Commission, 2021).

**Table 9:** Main results. Comparison between 2008 and 2021

	2008	2021
People with AD		
Total	167,700	239,600
Prevalence (per 1,000 aged 18+)	4.36	6.11
Informal caregivers of people with AD		
Total	141,617	202,102
Prevalence (per 1,000 aged 18+)	3.69	5.15
Provide until 16 caregiving hours/day (%)	42.87	59.04
1–5 h/day (%)	27.62	23.99
6–10 h/day (%)	37.09	35.04
11–16 h/day (%)	35.29	40.97
Provide more than 16 caregiving hours/day (%)	57.13	40.96
17–20 h/day (%)	10.36	8.70
21–24 h/day (%)	89.64	91.30
Annual informal caregiving hours		
Without censorship		
Per caregiver	5,263	5,281
Total (million hours)	745,330	1,061.3
With censorship		
Per caregiver	4,151	4,470
Total (million hours)	594.77	896,311,395
Valuation of informal caregiving hours		
Replacement method		
Per caregiver (€)	31,839 − 52,760	71,653 − 83,984
Total caregivers (million €)	4,509–7,472	14,316 − 16,850
With respect to GDP (%)	0.41–0.67	1.19–1.40
With respect to GDP per capita (%)	131.95- 218.65	281.00–329.30
With respect to average wage (%)	137–227	277–324
With respect to average retirement benefit (%)	326–540	502–588
Contingent valuation method (WTA)		
Per caregiver (€)	18,680 − 29,057	31,584 − 37,019
Total caregivers (million €)	2,645–4,115	6,310–7,427
With respect to GDP (%)	0.24–0.24	0.52–0.62
With respect to GDP per capita (%)	77.41–120.42	123.90–145.20
With respect to average wage (%)	80–125	59–143
With respect to average retirement benefit (%)	191–297	106–259

For the valuation of caregiving hours, we report the lower and upper interval obtained considering censored or uncensored hours

Estimations for 2008 obtained from Peña-Longobardo and Oliva-Moreno (2015)

Average wage (2008): 23,252 €/year

Average retirement benefit (2008): 9,774 €/year

### Multivariate analysis

Table 10 shows the regression analysis for the monetary valuation of informal care. Six regressions were carried out, three for censored hours and three for uncensored hours, in each case considering WTP (€3.3/hour), WTA (€6.9/hour) and replacement (€15.66/hour). We corroborate the relevance of the degree of dependence as an element of synthesis of the informal caregiver’s involvement in the basic and instrumental tasks of daily life. Considering that the (net) salary of a household employee in 2021 was €1,074.27^7^, we observe that monetary value of informal care provided in one week to a severe dependent represents between 0.57 (WTA) and 31.46 (replacement) the monthly wage of a domestic employee, and in the case of a highly dependent person, the value of informal care provided in one week is more than three times the monthly wage of a domestic employee (replacement). In addition, we highlight the following results: (i) a higher monetary valuation for female caregivers (between €2 and €6.15) and caregivers who have not completed primary education (between €24 and €122), (ii) a lower valuation for caregivers who have completed higher education (between €22 and €107). If the monetary valuation of informal care hours is in line with future demographic (and social) trends, then long-term care policies should internalise these effects when designing both benefits and the amount (intensity) of benefits.

**Table 10:** Regressions for the monetary value of weekly informal caregiving hours. OLS estimates

	Censored hours max. 16 h/day			Censored hours max. 24 h/day		
	WTP (3.3 €/hour)	WTA (6.9 €/hour)	Replacement (15.66 €/hour)	WTP (3.3 €/hour)	WTA (6.9 €/hour)	Replacement (15.66 €/hour)
**Carerereceiver**						
Men	13.06***	26.59***	64.24***	20.76***	42.33***	102.82***
	(2.42)	(3.39)	(5.33)	(3.71)	(5.21)	(8.18)
Age	-0.35***	-0.71***	-1.71***	-0.35***	-0.71***	-1.71***
	(0.05)	(0.08)	(0.12)	(0.07)	(0.10)	(0.16)
Moderate dependent	122.19***	271.49***	649.03***	195.76***	456.38***	1145.51***
	(2.78)	(3.90)	(6.13)	(4.28)	(6.00)	(9.42)
Severe dependent	254.72***	608.46***	1563.77***	398.16***	976.29***	2513.68***
	(3.12)	(4.39)	6.89)	(4.81)	(6.74)	(10.59)
Highly dependent	555.49***	1392.27***	3783.66***	734.71	1890.05***	5197.70***
	(3.00)	(4.21)	(6.61)	(4.61)	(6.46)	(10.15)
**Informal caregiver**						
Women	2.05***	4.10***	6.16***	2.45***	4.91***	7.36***
	(0.42)	(0.39)	(1.32)	(0.71)	(0.20)	(1.16)
Age	0.11	0.21	0.51	0.14	0.28	0.68
	(0.02)	(0.03)	(0.04)	(0.04)	(0.05)	(0.08)
Incomplete primary education	24.55***	50.10***	122.00***	36.95***	75.65***	185.72***
	(7.98)	(11.20)	(17.60)	(12.23)	(17.18)	(27.02)
Primary education or equivalent	14.90	30.33	73.38***	16.73	34.09	82.56***
	(7.66)	(10.76)	(16.91)	(11.75)	(16.51)	(25.97)
1st stage secondary education	2.87	5.83	13.99	6.14	12.47	29.97
	(7.62)	(10.71)	(16.83)	(11.69)	(16.44)	(25.84)
Baccalaureate studies	-10.33	-20.91**	-49.69***	-24.53**	-49.43***	-116.26***
	7.98)	11.20)	17.60)	12.23)	17.18)	27.02)
Intermediate vocational education	-12.53	-25.33***	-60.10***	-25.87**	-52.12***	-122.48***
	(8.32)	(11.68)	(18.36)	(12.76)	(17.93)	(28.20)
Higher vocational education	-18.36***	-37.07***	-87.58***	-32.42***	-65.21***	-152.48***
	(8.39)	(11.78)	(18.53)	(12.87)	(18.08)	(28.44)
University education or equivalent	-22.52***	-45.41***	-106.97	-36.55***	-73.43***	-171.15***
	(7.81)	(10.97)	(17.23)	(11.97)	(16.82)	(26.45)
Married	-11.28***	-22.80***	-54.16***	-12.28***	-24.83***	-58.94***
	(2.71)	(3.80)	(5.97)	(4.14)	(5.82)	(9.14)
Widow	3.99	8.11	19.47	10.88	22.13	53.39
	(5.62)	(7.90)	(12.40)	(8.63)	(12.12)	(19.05)
Separated	5.82	11.82	28.42	3.99	8.11	19.47
	(7.24)	(10.18)	(15.98)	(11.09)	(15.59)	(24.51)
Divorced	7.71	15.68***	37.75***	11.91	24.21***	58.46***
	(4.90)	(6.87)	(10.79)	(7.49)	(10.53)	(16.55)
**Size of municipality of residence**						
50.000-100.000 inhabitants	13.20***	26.88***	64.94***	18.85***	38.42***	93.20***
	(3.63)	(5.11)	(8.02)	(5.59)	(7.85)	(12.32)
20.000–50.000 inhabitants	13.56***	27.60***	66.70***	16.49***	33.58***	81.32***
	(3.49)	(4.91)	(7.69)	(5.35)	(7.51)	(11.79)
10.000–20.000 inhabitants	17.76***	36.18***	87.69***	27.03***	55.20***	134.64***
	(3.36)	(4.73)	(7.41)	(5.17)	(7.25)	(11.39)
Less than 10.000 inhabitants	23.77***	48.50***	118.04	31.22***	63.83***	156.11***
	(2.78)	(3.90)	(6.13)	(4.27)	(6.00)	(9.42)
Constant	210.37***	447.69***	922.49***	255.81***	549.28***	1515.09***
	(8.88)	(12.48)	(19.62)	(13.62)	(19.13)	(30.10)
N	427	427	427	427	427	427
R2	0.16	0.16	0.16	0.13	0.13	0.13
F	38.89	32.97	33.89	31.43	28.03	31.33
p	0.0000	0.0000	0.0000	0.0000	0.0000	0.0000

Omitted variables: caregiver characteristics (cannot read or write, single), carereceiver characteristics (not eligible), provincial capitals and municipalities with more than 100,000 inhabitants. Estimates obtained using sampling weights. Robust estimates

## Discussion and conclusions

In this study, we have estimated that the number of informal caregivers of people with AD in Spain in 2021 amounted to more than 200,000 people. The average number of weekly caregiving hours per caregiver ranges between 88 h (data censored at a maximum of 16 h of daily caregiving) and 104 h (uncensored data). In annual terms, individual average informal caregiving hours range between 4,586 and 5,430 (censored and uncensored data, respectively), and when scaled up to population level amount to between 927 and 1,097 million hours of care, mainly concentrated in people with a high degree of dependency.

The monetary valuation of informal caregiving time results in an estimate ranging from €71,653 to €83,984 per year and caregiver, using the replacement method (time values with and without censoring, respectively). The values estimated using the contingent valuation techniques are significantly lower, ranging between €15,183-€17,796 using WTP (with and without censoring, respectively) and between €31,584-€37,019 using WTA (with and without censoring, respectively). Scaling up to population terms, the total value obtained ranges from €14,316 to €16,850 million using the replacement method, which is equivalent to 1.2–1.4% of GDP for the same year and represents 148-174% of the entire SAAD budget.

Before commenting on the implications of these results, one should note that the range of variation in our estimates is wide, depending on the type of valuation technique used. For this reason, it is advisable to use more than one technique, where possible, since the interpretation and usefulness of the results obtained for policy design will depend on the objectives pursued by the policies. In the case of the replacement method, the value provided is the shadow price of the closest substitute. In our case, this would be the cost of one hour of home-help service, which includes both a part of personal care and a part of help with household chores. Contingent valuation methods, on the other hand, provide people’s valuation of a given good or service. In the case of receiving an improved good or service, the aim is to reveal the monetary valuation through the users’ WTP for it, so that their welfare would remain unchanged after receiving the service and making the payment. In case the good or service is withdrawn, the aim is to reveal the monetary amount that users should receive (WTA) in order to keep their welfare unchanged. Although traditional models postulate that the differences between the values obtained from the elicitation of the WTP and the WTA should be small, numerous empirical studies indicate significant differences between these observed values [74, 83]. For this reason, it seems appropriate, whenever possible, to adopt both points of view. In addition, although it has been pointed out that WTA seems more appropriate for assessing informal care, WTP is the technique most commonly used in practice [37, 54]. In this case, the valuation could serve as a minimum or conservative threshold for estimating the value of informal care from the perspective of caregivers.

Contingent valuation methods, on the other hand, provide people’s valuation of a given good or service. In the case of receiving an improved good or service, the aim is to reveal the monetary valuation through the users’ WTP for it, so that their welfare would remain unchanged after receiving the service and making the payment. In case the good or service is withdrawn, the aim is to reveal the monetary amount that users should receive (WTA) in order to keep their welfare unchanged. Although traditional models postulate that the differences between the values obtained from the elicitation of the WTP and the WTA should be small, numerous empirical studies indicate significant differences between these observed values [74, 83]. For this reason, it seems appropriate, whenever possible, to adopt both points of view. In addition, although it has been pointed out that WTA seems more appropriate for assessing informal care, WTP is the technique most commonly used in practice [37, 54]. In this case, the valuation could serve as a minimum or conservative threshold for estimating the value of informal care from the perspective of caregivers.

Both methods have their strengths, but also limitations. For this reason, whenever possible, more than one valuation tool should be used in the estimation of informal care. In the case of the replacement method, an implicit assumption is that informal care and professional care are perfect or near-perfect substitutes. Therefore, the performance by a formal caregiver of the tasks normally performed by the informal caregiver would result in no loss or gain of efficiency or quality. However, on the one hand, there are personal care tasks for which the training received by professional carers may invalidate this assumption. On the other hand, the affective relationship between informal carer and person cared-for also prevents the assumption of perfect substitution of informal care by professional care. Likewise, this method does not take into account the non-monetary opportunity costs of informal caregiving [4]. In addition, many caregivers need to devote more time to caring for people with AD as the disease progresses, often resulting in a withdrawal from their social networks [35]. Nor does it consider the positive effects derived from being a caregiver, i.e., maintaining the dignity and self-esteem of the person cared for, development of new skills and abilities, and the opportunity to nurture their relationship with the person they care for, knowing that the care recipient enjoys the fact that care is provided by that particular caregiver [30, 42, 68, 85]. With regard to contingent valuation, first, the literature shows us that the values of WTA are higher than those of WTP, when, under the Hicksian welfare theory, in a context of absence of uncertainty and with perfect information, the values of WTP and WTA should converge [56]. These differences can be explained within the standard neoclassical framework (asymmetry of the income effects; budgetary restriction; risk aversion). However, these discrepancies are also understandable under the alternative framework of behavioural models, using concepts such as reference dependence and loss aversion [43, 84]. Additionally, in the case of contingent valuation, there are special circumstances to be taken into consideration, such as the possible presence of protest responses or strategic biases in the responses [11]. These considerations show that, whenever possible, more than one valuation tool should be used in the estimation of informal care.

Back to our results, we identify a sharp growth in the number of caregivers of people with AD, which increased by 42.7% between 2008 and 2021. One of the possible reasons for this evolution is the ageing of the population. According to data from the Spanish National Institute of Statistics, the population aged 65 and over was 7.5 million in 2008 (16.4% of the population). In 2021 it rose to 9.3 million (19.7% of the population), representing an increase of 24.2% during that period. In addition, the increase in the population aged 85 and over rose by 74.4% between 2008 and 2021. Although AD is not an inevitable consequence of ageing, its prevalence is strongly concentrated in the older population [24, 50, 66, 69].

Another reason that might explain the observed results could be an improvement in the diagnosis of AD in the framework of the National Health System. Although this reason is hypothesized and there is no solid evidence in Spain to support it, both the scientific literature [66, 78] and the Comprehensive Plan for Alzheimer’s Disease and other Dementias (2019–2023) point out that this disease is underdiagnosed, especially in mild cases [51], and that improving early diagnosis is one of the general objectives of the Plan. Whether the improvement in medical services has included an improvement in the diagnosis of this disease is a question that will have to be tested in future scientific work.

Another consideration is that the sharp increase in the number of caregivers between 2008 and 2021 occurred in a context of development and deployment of publicly funded professional LTC (SAAD). The Dependency Law (approved in December 2006) favoured the creation of new places in facilities by improving funding and access to these places for lower-income groups^8^. According to the Institute for the Elderly and Social Services (IMSERSO), an official body under the aegis of the Ministry of Social Affairs (which has assumed various names over the last decade), in 2010 Spain had 368,805 places in facilities. This represents a sharp increase with respect to previous years, since in 2001 the number of places was 239,761, distributed among 4,800 centres^9^. Since then, the growth has been continuous, but more moderate in recent years, reaching 397,743 places in 2021, distributed among 5,485 centres^10^. This suggests that the number of people with AD living in facilities should have increased between 2008 and 2021 in Spain, but there is hardly any official information or any scientific publications that shed light on the health status and disease profile of people living in facilities in Spain. EDAD-08 had a special module, carried out in facilities, that estimated the number of people with dementia of Alzheimer’s type, and living in residential care, at 39,134. EDAD-2020 has published in May 2024 a comparable information about the health status of people living in facilities that indicate that 48.783 people with dementia of Alzheimer’s type lived in residential care in 2021. However, more research is needed to find out whether these figures are close to reality or underestimate the number of people with AD living in facilities.

In any case, given that an estimated 700,000 people in Spain suffer from this disease [51] and that only 200,000 people live in their own homes and require personal care, it is worth asking what weight of this difference is due to (i) people with AD who live in facilities; (ii) people with AD who live in their own homes and only receive professional care; (iii) people diagnosed with a mild degree of disease development who do not require personal care at this time (professional or informal); (iv) people who have not yet received a diagnosis.

Closely related to the growth in the number of people with AD and receiving care, the evolution of the figures for the value of care time is remarkable. Considered in relation to GDP, the value of care time was established in 2008 [59] in a range of values from 0.41 − 0.67% of GDP (replacement method) to 0.24% (contingent valuation - WTA). By contrast, in this paper the value of informal care time estimated in 2021 ranges between 1.2 and 1.4% of GDP (replacement method) and between 0.52 and 0.62% (contingent valuation - WTA). This highlights the concerning growth in family resources devoted to caring for people with AD, especially if we consider that forecasts point to an increasing prevalence of the disease associated with population ageing, unless preventive programmes and therapeutic innovations slow down this progression.

In connection with the above point, it is important to note that despite the increase in professional resources arising from the development of the SAAD between 2008 and 2021, the time spent on informal care by each informal carer was very similar in 2021 to that spent in 2008. One interpretation of this result is that, in the case of people with AD residing at home, professional care and informal care seem to behave as complementary services rather than as substitutes for each other [7, 12, 23, 40, 45, 48, 71]. However, another interpretation, compatible with the previous one, is that a greater presence of home care services could imply a change in the type and intensity of tasks performed by informal caregivers [40, 90]. Unfortunately, with the information provided by the EDAD-2020 we cannot test such hypotheses. In any case, it does not appear that the development of the Spanish LTC system has resulted in a reduced burden of care, meaning the almost complete availability of the time of a large proportion of the main caregivers in the case of Alzheimer’s disease.

Another relevant aspect that we would like to highlight is the large number of hours of care provided to people with AD which, in principle, would not reach the minimum score required by the dependency scale applied in Spain. To respond to this apparent contradiction, it should be pointed out that being categorised as ineligible is not equivalent to assuming that these persons have no degree of dependence. Royal Decree 174/2011, of February 11th, 2011, approving the Dependency Scale states: “*it should be noted that a score of less than 25 points in the BVD determines exclusively that the person does not have a situation of dependency with a recognized degree for the purposes of Law 39/2006*,* of December 14*,* 2006*,* which does not always imply a situation of full independence or total autonomy*”. So being categorised as “noneligible” does not imply that these people do not require care to be able to cope with their basic activities of daily living, even in the early stages of the disease. In this context, [10] investigated the relationship between cognitive function and dependence on care before patients reached a severe stage of the disease. They found that 69% of patients in the early stage of Alzheimer’s disease required more than 12 h of supervision per day. This is explained by the fact that sometimes a stage of dependency is reached quite early in the disease, when caregivers decide that patients can no longer be safely alone. This may result in a significant amount of time spent supervising the person.

This paper shows a part of the social opportunity cost of AD, but the results should be interpreted as a conservative approximation, given the following reasons: first, we focused on informal care, without valuing other relevant resources such as health and professional non-health care. Secondly, we have only identified caregiving hours of the main caregiver. However, it is common for there to be more than one caregiver in the affective environment of people with AD. Thirdly, the significant differences between the number of people with AD and residing at home (whether or not they receive care), according to our main data source (EDAD-2020), and the prevalence figures estimated for Spain.

Other limitations to be taken into account are that, although EDAD-2020 is an excellent survey, with rich and varied information, it is not aimed exclusively at people with AD. For this reason, it is difficult to compare the results with those of other studies that have used specific questionnaires such as the RUD- Resource Utilization in Dementia [2]. In addition, the EDAD-2020 directly asks about care time, but does not give details of the time spent on each task. It would have been interesting to know the distribution of care time and to be able to control for situations of joint production, in which the caregiver may be spending time supervising or accompanying the patient while enjoying a leisure activity (reading, watching TV, for example) or doing another task at the same time. Likewise, we have no information about the activities that the carer might have carried out. Specifically, about whether caregivers have had to give up part or all of their working time, or whether they have done so temporarily or permanently. Thus, it has not been possible to carry out a valuation of time using the opportunity cost method. A final detail to be mentioned is that the EDAD-2020 is a cross-sectional survey. The availability of longitudinal data could have made it possible to answer additional questions relating to: (i) changes in care time during the progression of the disease, controlling for unobservable elements of heterogeneity, (ii) analyses of the degree of complementarity or substitutability of professional and informal care (iii) changes in the profile of informal caregiving and (iv) transitions when the person with AD changes from home care to institutionalisation in a nursing home for people with AD.

This study highlights the enormous social value of informal care for people with AD. At the same time, it highlights the large number of weekly and annual hours supported by caregivers. Although other research has identified positive aspects related to caregiving [4, 20, 29, 30], it is also true that the high intensity of caregiving time and caregiving over long periods of time (years) results in an overload for caregivers that can affect their health, work status, family relationships - in short, their well-being [4, 39, 63]. In this sense, a key aspect of LTC policies should be to understand the needs of carers [80] and to provide the support they need in different dimensions (information, training, respite care, professional care). In Europe and around the world, there are large differences in caregiver support policies [16]. However, given existing forecasts of the growth in the number of people with AD [51], and of demographic and social changes that may limit the availability of informal care [9], there needs to be a major shift in LTC policies to provide an integrated view of professional and informal resources and to explicitly consider the social value of the latter.

Finally, we would like to point out that the results of our exercise, although confined to the Spanish context, may be of importance for other environments, especially in the case of countries that will face population ageing processes in the coming decades. Although the results and conclusions are not directly extrapolable due to the differences between countries in demographic and epidemiological evolution, in LTC systems, or to cultural or economic differences, without exhausting the list, conducting similar exercises in other countries, and comparing them, could generate information of undoubted value for the design and implementation of policies aimed at complementing professional and informal care in order to improve the quality of life both of the people who suffer from this terrible disease and of the people who are responsible for their care and attention.

## Electronic supplementary material

Below is the link to the electronic supplementary material.

Supplementary Material 1

## Appendix A

**Table A1:** Valuation of informal care using contingent valuation method

	Valuation of annual caregiving hours for all informal caregivers			
	Censored hours (max. 16 h/day)		Not censored hours (max. 24 h/day)	
	WTP	WTA	WTP	WTA
**Euros**				
NE	21,094	23,921	26,691	30,268
MD	22,846	25,907	28,282	32,073
SD	24,720	28,033	29,405	33,346
HD	28,899	32,772	32,370	36,709
Average	25,662	29,101	30,078	34,109
**Average valuation with respect to average wage (2021)**				
	99%	112%	69%	116%
**Average valuation with respect to average retirement benefit (2021)**				
	180%	204%	125%	211%
**Percentage with respect to per capita GDP (2021)**				
NE	82.7%	93.8%	104.7%	118.7%
MD	89.6%	101.6%	110.9%	125.8%
SD	96.9%	109.9%	115.3%	130.8%
HD	113.3%	128.5%	126.9%	144.0%
Average	100.6%	114.1%	118.0%	133.8%
	**Valuation of annual caregiving hours for all informal caregivers**			
	**Censored hours (max. 16 h/day)**		**Not censored hours (max. 24 h/day)**	
	**WTP**	**WTA**	**WTP**	**WTA**
NE	973.3	1,103.7	1,231.5	1,396.6
MD	603.5	684.3	747.1	847.2
SD	1,140.6	1,293.5	1,356.7	1,538.6
HD	2,409.8	2,732.8	2,699.3	3,061.0
All	5,127.1	5,814.3	6,034.6	6,843.4
**Percentage with respect to GDP (2021)**				
NE	0.08%	0.09%	0.10%	0.12%
MD	0.05%	0.06%	0.06%	0.07%
SD	0.09%	0.11%	0.11%	0.13%
HD	0.20%	0.23%	0.22%	0.25%
All	0.42%	0.48%	0.50%	0.57%
**Percentage with respect to total expenditure of the SAAD (2021)**				
NE	10.0%	11.4%	12.7%	14.4%
MD	6.2%	7.1%	7.7%	8.7%
SD	11.8%	13.3%	14.0%	15.9%
HD	24.8%	28.2%	27.8%	31.5%
All	52.8%	59.9%	62.2%	70.5%

WTP: willingness to pay; WTA: willingness to accept

WTP (Garrido-García et al., 2015) and WTA (Oliva-Moreno et al., 2019)

NE: non eligible, MD: moderate dependent; SD: severe dependent; HD: highly dependent

GDP (2021): 1,222,290 million €. Source: INE (*INEbase / Economía /Cuentas económicas /Contabilidad nacional trimestral de España: principales agregados (CNTR) / Resultados*)

GDP per capita (2021): 25,498€

Expenditure of the SAAD (2021): 9,704,647,135.52 €. Source: *7e8ae15d-0915-ab4e-6c46-ccb0f1f3e8e2 (imserso.es)*

**Table A2:** Valuation of annual caregiving hours of informal caregiver receiving cash subsidy

	Contingent valuation method			
	Censored hours (max. 16 h/day)		Not censored hours (max. 24 h/day)	
	WTP	WTA	WTP	WTA
MD	23,074	26,166	28,565	32,393
SD	25,214	28,594	30,287	34,346
HD	29,477	33,428	33,018	37,443
**Percentage with respect to minimum cash subsidy (2021)**				
	**Contingent valuation method**			
	**Censored hours (max. 16 h/day)**		**Censored hours (max. 16 h/day)**	
	**WTP**	**WTA**		
MD	8.0%	7.0%	6.4%	5.7%
SD	12.8%	11.3%	10.6%	9.4%
HD	15.8%	13.9%	14.1%	12.4%
**Percentage with respect to minimum cash subsidy (2021)**				
	**Contingent valuation method**			
	**Censored hours (max. 16 h/day)**		**Censored hours (max. 16 h/day)**	
	**WTP**	**WTA**	**WTP**	**WTA**
MD	8.0%	7.0%	6.4%	5.7%
SD	9.6%	8.5%	8.0%	7.0%
HD	11.8%	10.4%	10.6%	9.3%

WTP: willingness to pay; WTA: willingness to accept

WTP (Garrido-García et al., 2015) and WTA (Oliva-Moreno et al., 2019)

MD: moderate dependent; SD: severe dependent; HD: highly dependent

Minimum cash subsidy: 1,836 €/year (MD); 2,419.08 €/year (SD); 3,488.76 €/year (HD)

Maximum cash subsidy: 1,836 €/year (MD); 3,225.48 €/year (SD); 4,651.68 €/year (HD)

**Table A3:** Valuation of annual caregiving hours of informal caregiver when AD patient receives public home care

	Contingent valuation method (€/year)			
	Censored hours (max. 16 h/day)		Not censored hours (max. 24 h/day)	
	WTP	WTA	WTP	WTA
MD	16,084	18,240	18,761	21,276
SD	25,012	28,365	33,046	37,475
HD	24,736	28,051	31,919	36,197
**Cost of public home care with respect to valuation of informal care (2021)**				
	**Contingent valuation method**			
	**Censored hours (max. 16 h/day)**		**Censored hours (max. 16 h/day)**	
	**WTP**	**WTA**	**WTP**	**WTA**
MD	67.76%	59.76%	58.10%	51.23%
SD	24.79%	21.86%	18.77%	16.55%
HD	10.64%	9.38%	8.24%	7.27%

WTP: willingness to pay; WTA: willingness to accept

WTP (Garrido-García et al., 2015) and WTA (Oliva-Moreno et al., 2019)

MD: moderate dependent; SD: severe dependent; HD: highly dependent

Public price of home care hour: 15.66 €/hour

MD receive between 8 and 20 h/month; SD between 21 and 45 €/month; HD between 46 and 70 €/month. In order to calculate the public cost of home care according to the degree of dependency, we have assumed that the patient receives the average number of hours corresponding to his or her degree of dependency

**Table A4:** Valuation of annual caregiving hours of informal caregiver when AD patient receives attention in a public day centre

Contingent valuation method (€/year)				
	Censored hours (max. 16 h/day)		Not censored hours (max. 24 h/day)	
	WTP	WTA	WTP	WTA
MD	14,330	16,250	14,330	16,250
SD	13,959	15,830	15,324	17,378
HD	23,278	26,398	31,876	36,148
**Cost of public day centre with respect to valuation of informal care (2021)**				
	**Contingent valuation method**			
	**Censored hours (max. 16 h/day)**		**Censored hours (max. 16 h/day)**	
	**WTP**	**WTA**		
MD	64.96%	57.29%	64.96%	57.29%
SD	66.69%	58.81%	60.75%	53.57%
HD	39.99%	35.26%	29.20%	25.75%

WTP: willingness to pay; WTA: willingness to accept.

WTP (Garrido-García et al., 2015) and WTA (Oliva-Moreno et al., 2019).

MD: moderate dependent; SD: severe dependent; HD: highly dependent.

Public price of place in day centre (2021): 9309,21 €/year.

## Footnotes

1 Source: INE, EDAD-2020 methodology meto_edad_2020.pdf (ine.es).

2 Sources INE *INEbase / Economía /Cuentas económicas /Contabilidad nacional trimestral de España: principales agregados (CNTR) / Resultados*; *INEbase / Mercado laboral /Salarios y costes laborales /Encuestas de estructura salarial / Últimos datos*; Ministry of Labour and Social Economy: *Avance Anuario de Estadísticas 2021. Ministerio de Trabajo y Economía Social (mites.gob.es)*)

3 Source: IMSERSO: Report on the evolution of dependency funding; https://imserso.es/documents/20123/107160/inf_evol_2020_2021.pdf/7e8ae15d-0915-ab4e-6c46-ccb0f1f3e8e2?t=1663600983298.

4 Source: https://www.mdsocialesa2030.gob.es/derechos-sociales/inclusion/docs/estudio_evaluacion_saad_completo.pdf.

5 Source: *BOE-A-2013-13811 Real Decreto 1051/2013*,* de 27 de diciembre*,* por el que se regulan las prestaciones del Sistema para la Autonomía y Atención a la Dependencia*,* establecidas en la Ley 39/2006*,* de 14 de diciembre*,* de Promoción de la Autonomía Personal y Atención a las personas en situación de dependencia.*

6 Source: IMSERSO 8f175b2f-016d-17e2-bbb1-ea46141ea095 (imserso.es).

7 Source: Tabla Salarial Empleadas de Hogar 2021 - Aiudo Blog.

8 However, given that there has also been an increase in resources for telecare services, home help and day centres, an increase in the number of people cared for at home cannot be ruled out.

9 https://imserso.es/el-imserso/documentacion/estadisticas/servicios-sociales-dirigidos-personas-mayores-espana/servicios-sociales-dirigidos-personas-mayores-espana-diciembre-2010/servicio-atencion-residencial.

10 https://imserso.es/el-imserso/documentacion/estadisticas/servicios-sociales-dirigidos-a-personas-mayores-en-espana-diciembre-2021.
